# The Potential of Apulian Olive Biodiversity: The Case of Oliva Rossa Virgin Olive Oil

**DOI:** 10.3390/foods10020369

**Published:** 2021-02-09

**Authors:** Giacomo Squeo, Roccangelo Silletti, Giacomo Mangini, Carmine Summo, Francesco Caponio

**Affiliations:** 1 Food Science and Technology Unit, Department of Soil Plant and Food Sciences, University of Bari “Aldo Moro”, Via Amendola 165/A, 70126 Bari, Italy; roccangelo.silletti@uniba.it (R.S.); carmine.summo@uniba.it (C.S.); francesco.caponio@uniba.it (F.C.); 2 Institute of Biosciences and Bioresources (IBBR), National Research Council (CNR), Via Amendola 165/A, 70126 Bari, Italy; giacomo.mangini@ibbr.cnr.it

**Keywords:** olive landrace, ripening, harvest season, antioxidants, minor compounds, oil quality

## Abstract

In this study, the drupes and virgin olive oils extracted from the Oliva Rossa landrace are characterized. Oliva Rossa is an old landrace part of the autochthonous Apulian olive germplasm for which only few data have been reported till now. During the study, the maturity patterns of the drupes had been followed. Four samplings per year were planned, one every 14 days starting from the middle of October. The pigmentation index, the oil content and the total phenolic content of the drupes were measured. Simultaneously, virgin olive oils were extracted at the lab scale and analyzed for the fatty acid composition, the basic quality parameters and the content of minor compounds. The pigmentation pattern of the drupes was different among the years and, despite this trend, at the third sampling time the stage of maximum oil accumulation was always over. The extracted virgin olive oils had a medium to high level of oleic acid. With colder temperatures, a higher level of monounsaturated fatty acids, oleic/linoleic ratio and antioxidants was observed. The phenolic profile was dominated by 3,4-DPHEA-EDA and *p*-HPEA-EDA while the volatile profile by (*E*)-2-hexenal and 3-ethyl-1,5-octadiene.

## 1. Introduction

Extra virgin olive oil (EVOO) is one of the most renown advocate of the Mediterranean diet all over the world, representing its principal source of fat [1,2,3]. Its worldwide appreciation is linked to key features that include its hedonistic aspects together with the nutritional and the healthy ones [4,5,6,7,8]. However, despite the great importance gained, quite often the discussion about EVOO is generic and the “extra virgin olive oil” class becomes a huge container in which the single features and particularities of different EVOOs are lost. This approach may lead unfamiliar and also traditional consumers to the misconception that all the EVOOs are the same and also increase the perception of EVOO as a commodity [9]. In last decades, this behavior has been even enhanced by the fact that the majority of the virgin olive oil (VOO) is produced from few cultivars together with the development of super-intensive cultivation [10], despite the huge degree of olive biodiversity at disposal worldwide. As proof of this, it is reported that around 9% of the total Spanish varieties accounted for 96% of the olive-growing surface area in Spain; in Italy, 80 varieties out of 538 accounted for 90% of the total area; and in Greece, only 3 cultivars accounted for about 90% of the total area [10].

However, in contrast with such trend, actions have been taken toward the characterization, differentiation and valorization of monovarietal olive oils and olive biodiversity. This is proven by the rise in studies aimed at the valorization of olive biodiversity at the national [11,12,13,14] or local scale [15,16,17,18].

The safeguard and valorization of biodiversity has become a crucial matter in national and international policies [19]. Biodiversity is the key for a resilient and sustainable agriculture and represents one of the most important heritage to be preserved. One of the most important tools in this sense, adopted by the EU about two decades ago, is the designation of origin (DO) of food products [20]. DOs represent and implicit link with specific territories and, indeed, with their genetic resources.

In recent years, an increasing role of the olive variety and of the place of origin in driving the marketing strategies has been observed in the Italian olive oil market, even if the differentiation among EVOOs is still vertical and the market segmentation strongly based on habits [21]. Nonetheless, Cacchiarelli and colleagues [21] observed that EVOOs from local cultivars still get a less premium price, probably as a consequence of a lack of information on the consumers’ side. Hence, it appears clear that supporting biodiversity and local typical production goes through the knowledge and the communication to the consumers of the specificity of local products.

Italy has a large olive germplasm, estimated in over 500 accessions, including varieties, old local landraces and feral forms, which makes it the leading country for olive biodiversity [10,15,22]. In particular, old local landraces may represent a treasure at the disposal of farmers, producers, olive mills and sellers in the framework of a comprehensive valorization of the territory. Among these, “Oliva Rossa” represents an old landrace belonging to the autochthon Apulian olive germplasm [23].

Only few traces about the characteristics of this cultivar and of the derived VOOs could be found in the literature. According to the Italian National Review of Monovarietal Olive Oils [24] and the Olea database [25], Oliva Rossa is a synonym of “Oliastro” and is reported to be autochthonous to the Apulia region. Other synonyms of the landrace are “Lezze” in the area of Brindisi; “Olivastro del Gargano” in the area of Foggia; and “Olivasto di Conversano” in Bari province [23,25]. The old landrace is designed for oil extraction while no information about other purposes has been reported. The trees show a dense canopy and a medium vigor while the drupes are characterized by many lenticels, an elliptic shape and a low-to-medium weight and oil content [25]. Moreover, from the little information available, Oliva Rossa oils are characterized by almond, artichoke and cut-grass notes, among others [24], and the phenolic content is noteworthy, with a mean value of about 700 mg kg^−1^ (based on three samples from two different harvest crops). Today, Oliva Rossa could be considered basically an old olive landrace. It is planted only in small areas, in which it is still difficult to find representative plantations since the trees are dispersed in arable or in old orchards consociated with other tree fruits, such as almond or cherry.

The drupes are generally harvested together with modern olive cultivars for oil extraction. As a consequence, monovarietal oils from Oliva Rossa are more unique than rare.

Thus, in the framework of the valorization of the Apulian autochthonous olive germplasm, the aim of the study was to characterize and extend the knowledge about Oliva Rossa VOO. For the purpose, the maturity pattern of the drupes and the chemical characteristics of the relative VOOs were studied for four consecutive harvest seasons.

## 2. Materials and Methods

### 2.1. Plant Material, Sampling Plan and VOO Extraction

Drupes of the old olive landrace Oliva Rossa were harvested in four consecutive harvest seasons, from 2016/2017 to 2019/2020, in an olive tree field located in Putignano (Bari, Italy). Drupes were sampled from three different trees every 14 days (Sampling 1, S1; Sampling 2, S2; Sampling 3, S3; and Sampling 4, S4), starting around the middle of October (±one week) and, more in detail, two-weeks before the expected physiological maturity (defined as the half-veraison of the fruits). On the whole, around 10 kg of drupes were collected at each sampling time. For each sampling, the drupes were divided into 3 aliquots (around 3 kg each), representing the biological replicates. VOOs were extracted starting from the 2nd sampling (S2)—supposed to correspond to the physiological maturity—to the last (S4) by a lab scale plant made up of a hammer mill and a bask centrifuge [26]. On the whole, *n* = 9 VOOs were supposed to be extracted per each harvest season, 3 per each sampling point. Once extracted, the VOOs were sealed in glass bottles and stored in cold and dark conditions till the moment of analysis. Data about temperatures and rainfalls during the studied years were obtained from the Apulian civil protection [27] and reported in Figure S1. The weather station was about at 8 km from the orchard including the Oliva Rossa trees.

### 2.2. Drupes Analysis

The ripening degree of the fruits at each sampling time was calculated as the pigmentation index, Pi [28]. Pi ranges from 0 to 5 and is obtained by dividing the drupes into 5 classes taking into account the color of the skin and eventually the flash color. The moisture of the drupes was measured with an automatic moisture analyzer (Mod. MAC 110/NP, Radwag Wagi Elektroniczne, Radom, Poland) and used to express the results of the subsequent determinations on the basis of fruit dry weight. The total oil content was determined by using the Soxhlet apparatus and diethyl ether as a solvent. After extraction the solvent was removed, and the oil content was express as a percentage with respect to the fruit dry weight. For the extraction of total phenolic compounds, about 1 g of olive paste was mixed with 10 mL of a mixture of methanol/water (70/30) and 5 mL of hexane. Then the mixture was kept in agitation for 10 min and afterward centrifuged (SL 16R Centrifuge, Thermo Fisher Scientific Inc., Waltham, MA, USA) for 10 min at 4 °C at 3941× *g*. The hydro alcoholic phase was recovered and centrifuged again at 8867× *g* for 5 min. Finally, the methanolic phase was filtered (0.45 μm, VWR International, Center Valley, PA, USA) into an amber glass vial. The phenolic extracts were used for the determination of the total phenolic content (TPC) by means of the Folin-Ciocalteu reagent, as previously reported [29,30]. In brief, 100 µL of extract was mixed with the same amount of Folin reactive (Sigma-Aldrich Co. LLC, St. Louis, MO, USA) and, after 4 min, 800 µL of Na_2_CO_3_ (5% *w/v* in water) (Carlo Erba Reagents S.r.l., Cornaredo, Italy) were added. The final solution was heated at 40 °C for 20 min and after 15 min of cooling the absorbance at 750 nm was measured (Agilent Cary 60 spectrophotometer, Agilent Technologies, Santa Clara, CA, USA). If needed, proper dilution of the extract was carried out. Quantitation was achieved by means of an external calibration curve of gallic acid (*R^2^* = 0.998) and the results expressed as mg of gallic acid equivalents (GAE) per kg of dry weight.

### 2.3. VOO Analysis

The determination of fatty acid composition was carried out after sample transesterification with KOH 2N in methanol [31] by a GC (Agilent 7890A gas chromatograph, Agilent Technologies, Santa Clara, CA, USA) equipped with an FID detector (set at 220 °C) and an SP2340 capillary column of 60 m × 0.25 mm (i.d.) × 0.2 mm film thickness (Supelco Park, Bellefonte, PA, USA). The identification of each fatty acid was carried out by comparing the retention time with that of the corresponding standard methyl ester (Sigma-Aldrich, St. Louis, MO, USA). The amount of single fatty acids was expressed as area % with respect to the total area. Basic quality analyses of the VOOs (free fatty acids, FFA; peroxide value, PV; K_232_, K_270_) were carried out according to the official regulations [31]. VOO hydrophilic antioxidants were extracted as previously described for drupes with slight modifications. Basically, about 1 g of oil was mixed with 5 mL of methanol/water mixture and 2 mL of hexane. Then the extraction protocol, the Folin assay and the quantitative determination were done the same as previously reported (see Section 2.2). Pigments (chlorophylls and carotenoids) were measured spectrophotometrically (Agilent Cary 60 spectrophotometer, Agilent Technologies, Santa Clara, CA, USA), as reported in [32,33], respectively. Tocopherols were determined by RP-UHPLC-FLD (ThermoScientific, Waltham, MA, USA), as reported in previous work [28]. For the analysis of single phenolic compounds, phenolic extracts were obtained according to the procedure already described for the Folin assay with minor modifications. In detail, 5 g of sample were used with 2 mL of methanol/water mixture and 2 mL of hexane. Then, 250 µL of a 100 mg L^−1^ solution of gallic acid was added as internal standard for quantitation. The separation was obtained by RP-UHPLC-DAD (ThermoScientific, Waltham, MA, USA), as described in [34]. The identification was carried out by comparison of the retention times with those of pure standards and, if not available, with the literature data. Quantification was carried out on the signal recorded at 280 nm and the results were expresses as mg of gallic acid equivalent (GAE) per kg of oil. The phenolic profile was reported only for the harvest season 2017/2018.

The analysis of volatile compounds was carried out by means of HS-SPME-GC-MS, as reported in a previous work [35]. Briefly, for the extraction of volatiles, about 1 g of sample was sealed in a 20 mL vial after the addition of 100 µL of a 60 mg kg^−1^ solution of 1-octanol in purified olive oil as internal standard for quantification. Then, it was first conditioned at 40 °C for 2 min and, thereafter, the SPME fiber (50/30 μm DVB/CAR/PDMS; Supelco, Bellefonte, PA, USA) was exposed in the vial headspace for 20 min. The desorption was carried out directly in the GC-MS (Agilent 6850 series gas chromatograph coupled to a mass spectrometer Agilent 5975 series; Agilent Technologies, Santa Clara, CA, USA) injector at 250 °C for 2 min. The stationary phase was an HP-Innovax polar column. Volatile compounds were identified by comparison of their mass spectra with those in the NIST library. Only those with a match quality above 70% were considered. Results were expressed as mg of 1-octanol equivalents (OE) per kg of oil. The volatile profile was reported only for the harvest season 2017/2018.

### 2.4. Statistical Analysis

Each analysis was carried out at least in duplicate per each biological replicate (*n* = 3). One-way and two-way analysis of variance were carried out by means of Minitab 17 (Minitab Inc., State College, PA, USA) and significant differences were highlighted by Fisher’s LSD post-hoc test at α = 0.05.

## 3. Results

### 3.1. Sampling Issues

Along the four years of the current study, two harvest seasons (2016/2017 and 2018/2019) have been characterized by intense pest attacks and/or other agro-climatic issues, such as late frost, which destroyed almost all the production [36]. In these seasons, only two samplings (S1 and S2) out of the four planned were carried out. As a consequence, the discussion of the results will be mostly based on those obtained during the harvest seasons 2017/2018 and 2019/2020 although all the available results are reported.

### 3.2. Drupes Characteristics

**Figure 1:**
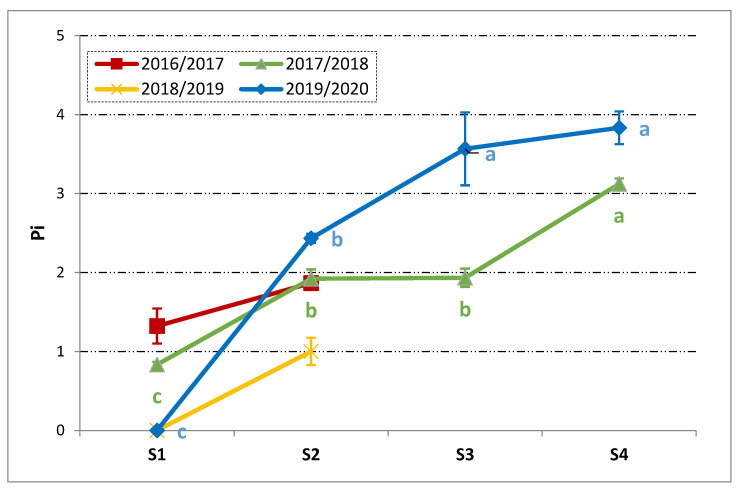
Trends in the Pi (mean ± SD, *n* = 3) of the Oliva Rossa drupes during ripening in four consecutive harvest seasons. Different letters for each harvest year indicate significant differences according to one-way ANOVA followed by Fisher’s LSD post-hoc test (α = 0.05). S1–S4: four subsequent samplings.

The ripening process of the drupes was measured as Pi and it is reported in Figure 1. As a first glance, it appears the maturity pattern was different among the harvest seasons. Depending on the year, the drupes started with different values of Pi. In 2018/2019 and 2019/2020, the drupes started totally green (Pi = 0) while they were slightly spotted in the case of 2016/2017 and 2017/2018 (Pi around 1, i.e., <50% black skin with white flash).

It is worthy to highlight that the first sampling (S1) fell in all the studied seasons around the middle of October. Following the evolution, the drupes reached a Pi of about 2 (≥50% black skin with white flash) in all the years, with exception of the season 2018/2019. This stage (Pi = 2) correspond to the stage commonly considered the physiological maturity (half-veraison of the fruits) and expected to be the stage in which the oil accumulation reached its maximum (i.e., technological maturity).

Thereafter, an important gap among the years 2017/2018 and 2019/2020 was recorded at S3. In the latter, the full maturity seemed almost reached and, from this point on, only a slight increase in Pi was recorded. Differently in 2017/2018, the Pi remained practically unchanged between S2 and S3 while the most significant jump was observed from S3 to S4. On the whole, the ripening process proceeded in a more regular way during the harvest season 2019/2020. 

As evidenced by Camposeo et al. [37], the observed differences are likely related to the climatic conditions and crop load, which, among other factors, strongly influence drupe ripening. In this regard, the lower temperatures recorded during the ripening period in 2017 with respect to 2019 (±2–3 °C less, Figure S1) could be responsible for the observed delay in the pigmentation pattern.

**Figure 2:**
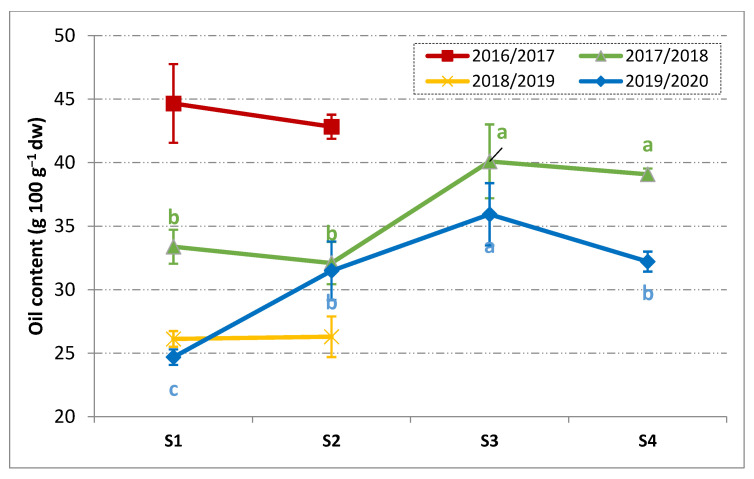
Oil content on dry weight (mean ± SD, *n* = 3) of the Oliva Rossa drupes during ripening in four consecutive harvest seasons. Different letters for each harvest year indicate significant differences according to one-way ANOVA followed by Fisher’s LSD post-hoc test (α = 0.05). S1–S4: four subsequent samplings.

From a technological point of view, it should be stressed how these differences were reflected in the drupes and in the oil characteristics. The evolution of the oil content during maturation is reported in Figure 2.

**Figure 3:**
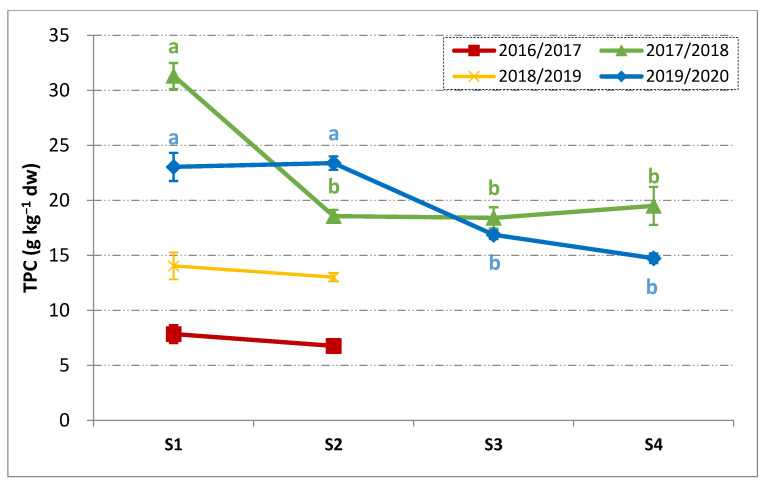
Total phenolic content (TPC) on dry weight (mean ± SD, *n* = 3) of the Oliva Rossa drupes during ripening in four consecutive harvest seasons. Different letters per each harvest year indicate significant differences according to one-way ANOVA followed by Fisher’s LSD post-hoc test (α = 0.05). S1–S4: four subsequent samplings.

The oil content at S1 ranged between 25 and 26 g 100 g^−1^ on dry weight (2018/2019 and 2019/2020) and goes up to 45 g 100 g^−1^ in 2016/2017. The season 2017/2018 lied in between the others, showing an initial oil content of 33 g 100 g^−1^. The very high oil content of the harvest season 2016/2017 should be considered as an exceptional case, confirming that such a season was an outlier with respect to the general pattern. In 2019/2020, the evolution of oil content was very similar to that of the Pi in Figure 1. Indeed, a regular accumulation of the oil was observed with a positive slope of about 5 g 100 g^−1^ each 14 days from S1 to S3. Thereafter, a small significant decrease in oil was reported. Such a slight decrease was likely linked to the natural variability of the sampled fruits among different trees and canopy positions. During 2017/2018, the most significant phase of oil accumulation occurred between S2 and S3, moving from 32 g 100 g^−1^ up to 40 g 100 g^−1^. Such an increase in oil was not reflected in the Pi, which was unaffected when moving from S2 to S3 (Figure 1). Afterward, the oil content remained constant. As a most important finding, in both the harvest seasons for which the full maturity pattern was followed, S3 corresponded to the maximum oil content. Unfortunately, the Pi did not reflect directly such a conclusion. In fact, at the half-veraison of the drupes (i.e., Pi = 2), generally considered as the moment of physiological maturity, the fruits could still be in the stage of intense oil accumulation, as observed in the 2019/2020 crop year. Figure 3 shows the evolution of the olives TPC.

During fruit ripening the TPC decreased, as is already known [38]. Further, in the harvest seasons 2016/2017 and 2018/2019, the negative effect of the pest attacks and late frost on the phenolic content of the drupes was easily observed. A high TPC was observed in 2017/2018, which was around 30 g kg^−1^ and about 25% less than in 2019/2020. The TPC of the Oliva Rossa drupes was similar to that reported for the Frantoio cultivar [38], while less than what was reported for the Sardinian cultivars [16]. Despite the initial gap at S1 between 2017/2018 and 2019/2020, the TPC tended to become similar during ripening and in particular at S3 the differences were negligible. The sharp TPC decrease from S1 to S2 in 2017/2018 could be linked to the abundant rainfalls recorded in October (Figure S1). Indeed, the tree water status is reported to be inversely correlated with the phenolic content [39]. It is noteworthy pointing out that, at S3, the phase of the more intense oil accumulation was already over (Figure 2) and the fruits also had comparable TPC. Thus, based on the available results, it would be reasonable considering S3 as the best moment for harvesting. Nonetheless the main issue of a feasible and reliable way of determining such a moment is still of concern considering that S3 corresponded to the stage in which the difference in the Pi was the biggest among the considered harvest seasons (Figure 1). The phenolic content of the drupes represents an important feature for the future VOOs and it should be considered that only 1–2% of the olives phenols moves into the oily phase during extraction [40], which may, in turn, depend on the fruit ripening [41]. 

### 3.3. VOOs Characteristics

Different features influence the definition of VOO quality. From a consumer point of view, the hedonistic aspects are relevant although also the nutritional ones may be of concern. The main impact on the nutritional score of VOO is ascribable to the FA (fatty acid) composition and particularly to the amount of oleic acid [42]. The phenolic compounds, together with other bioactive ones, also play a key role, as proven by the specific health claim adopted by the European Union [43]. On the other hand, while phenolic compounds are responsible for the organoleptic features of VOOs [44], it is not true for oleic acid. Finally, from a producer and seller point of view, compliance with the mandatory quality parameters defined by the EU law is of primary concern [31]. Starting from these considerations, and in the view of the valorization of the oils from Oliva Rossa, the VOOs were characterized for their fatty acid composition as well as the basic quality parameters and the minor compounds. Given the sampling issues related to the crop years 2016/2017 and 2018/2019—which may bias the comparison—only the results of the years 2017/2018 and 2019/2020 are reported and discussed. Table 1 reports the FA composition of the Oliva Rossa VOOs.

The palmitic (C16:0), stearic (C18:0), oleic (C18:1) and linoleic (C18:2) acids were the most abundant, as is typical in olive oils. Oleic acid, the main monounsaturated fatty acid, ranged from about 71% to just over 76%. The global mean value was roughly 73%, which is similar to the data reported elsewhere [24,25]. The crop year, the sampling time and the interaction among those factors showed an effect on the C18:1 content. The VOOs extracted during 2017/2018 had significantly higher values of C18:1 than those of 2019/2020. Further, while in 2017/2018 an increase in C18:1 was observed during ripening, the opposite was found in 2019/2020. The second most abundant fatty acid, linoleic acid, ranged between around 8% to 13.51%. A mean value of about 8% was reported in the Italian monovarietal oils databank [24]. 

Linoleic acid content was generally higher in 2019/2020 with respect to 2017/2018; also, a significant effect of the interaction between harvest year and sampling was observed. In particular, while in 2017/2018 a decrease in linoleic acid was reported during ripening, in 2019/2020 it was the opposite. The palmitic acid content showed a significant difference among the samples with an opposite trend in the studied harvest seasons. Indeed, the highest content was found at S4 in 2017/2018 while in S2 in 2019/2020. The stearic acid content was on average around 2.60% and it showed a regular decrease in content during 2017/2018, while again an opposite trend was observed in 2019/2020. Finally, other significant differences were observed for all the minor fatty acids. The content of some minor fatty acids (namely, miristic, arachic and gadoleic) in 2019/2020 oils deserves special attention, as they were close to the maximum value allowed by official regulations [31]. Altogether, it comes out that Oliva Rossa VOOs were richer in MUFA (total monounsaturated fatty acids) in 2017/2018 whilst richer in PUFA (total polyunsaturated fatty acids) in 2019/2020. Giving a clear explanation of the observed differences could be a difficult task considering the complex effect of single factors (genetic, environmental and agronomic), their interactions or independence, as reviewed by Inglese and others [39]. However, differences in the oleic, palmitic and linoleic acid content were mostly related to the course of temperatures during the year. In particular, lower temperatures could be correlated with a higher content of oleic acid and, on the opposite, with a lower content of palmitic and/or linoleic acids [39]. In the 2017/2018 crop season, the mean temperatures registered during fruits ripening were actually lower than those registered in the 2019/2020 season (Figure S1) and could justify the differences observed in terms of fatty acids composition. 

From a technological point of view, it is a fact that the different composition in fatty acids could in turn influence the oxidative stability of the product. In particular, values of O/L equals or higher than 7 have been suggested as an indication of good stability [45]. From Table 1, it could be observed that the ratio was different between the years and, in 2017/2018, it was always higher than 7. 

The results of the basic chemical analyses of the VOOs are reported in Table 2.

FFA is commonly the benchmark parameter for the vertical differentiation of olive oils into a specific product class (extra, virgin and lampante) [9]. The mean oil acidity ranged from 0.61% to 0.34%, being on average higher in the harvest season 2017/2018. The observed values could be considered quite high for freshly extracted oils. During maturation, a significant decrease in FFA was observed in 2019/2020. This decreasing trend was also reported by other authors [16]. Moving to the oxidation assessment, PV and K_232_ are generally considered markers of primary oxidation, while K_270_ is a marker of secondary oxidation products. Differences were highlighted considering both the harvest season and the sampling time. A specular trend was observed between the years. The oils extracted in 2019/2020 were affected by a higher extent of primary oxidation (PV and K_232_) but, on the average, by a less pronounced secondary oxidation (K_270_). This might be linked to the significantly higher amount of PUFA in oils from 2019/2020 and to the significantly lower value of O/L (Table 1), indices of a higher susceptibility to oxidation. 

On the whole, from Table 2, it could be stated that the Oliva Rossa VOOs still were classified as extra virgin olive oil (based on the reported parameters) till S3, which, in turn, corresponded to the maximum oil content (Figure 2). Obviously, these results cannot be generalized for all the VOOs obtained from this cultivar because the quality parameters are mostly influenced by the technological aspects of olive processing. Hence, it should be clear that such conclusions are related to the experimental conditions of the present study. 

The profile of the minor compounds of the oils is reported in Table 3. Minor compounds are fundamental molecules of VOO, which, in turn, influence its stability to oxidation, its nutritional and healthy aspects and its organoleptic features [44,46]. The oils had a high-medium content of TPC, ranging from about 800 to 350 mg kg^−1^. These results agree with the few available data in the literature [24,25]. The phenolic content decreased significantly during ripening, as is well known, in both the harvest seasons [37,47,48]. Moreover, the effect of the harvest season on the TPC was evident, with the 2017/2018 season having a general higher content with respect to the 2019/2020 season. The tocopherols content was also remarkable. The content of α-tocopherol decreased during ripening, without significant differences in 2017/2018. In 2019/2020, an outstanding content of α-tocopherol was reported at S2. The total amount of tocopherols followed the general trend reported for α-tocopherol considering that the latter represents roughly 90% of the total tocopherols in olive oils. The sum of the β- and γ-tocopherols ranged from about 6 to about 3 mg kg^−1^ with the highest significant value reached at S2 in 2019/2020.

Pigments are important compounds of olive oils, which affect the product stability and also could give useful information about fruit ripening and authenticity [46,49]. During ripening, the pigment content decreased although we observed a parabolic trend, especially considering the harvest season 2017/2018. Ripening is generally reported as the main source of variability in pigment content despite other factors [48,50], even if technology could also have an impact [51]. The ratio between chlorophylls and carotenoids (data not shown) was always very close to unity, as reported in other studies and suggested for authenticity purposes [49].

The profile of the phenolic compounds by HPLC for the harvest season 2017/2018 is reported in Table 4. In Figure S2, the relative chromatograms can be observed.

The identified compounds were those commonly found in VOOs, belonging to the classes of phenolic alcohols (3,4-DHPEA, *p*-HPEA), phenolic acids (vanillic and syringic acids), flavonoids (luteolin and apigenin), lignans (pinoresinol) and, most importantly, secoiridoid derivatives (3,4-DHPEA-EDA, *p*-HPEA-EDA and *p*-HPEA-EA). Phenolic acids were found in very little amounts, according to the literature data [44]. 

Similarly, a minor contribution of phenolic alcohols was observed. It is known that phenolic alcohols originate from the more complex secoiridoid moieties mostly during VOO storage [44]. The di-aldehydic forms of 3,4-DHPEA and *p*-HPEA were the most abundant phenols in all the maturity stages, followed by a remarkable amount of pinoresinol. At the first sampling (S2), the oils showed the highest significant content of almost all the identified phenolic compounds. During ripening, the profile changed with a sharp reduction in 3,4-DHPEA-EDA and a less pronounced decrease in *p*-HPEA-EDA. At S3, the oils were characterized on one hand by the significantly higher amount of luteolin and 3,4-DHPEA-EDA-CARB while on the other by the significant lower content of *p*-HPEA-EDA and pinoresinol. At S4, the lowest significant amount of phenolic compounds was observed. Some phenols showed a parabolic trend during ripening, having a maximum or minimum content in correspondence to the technological optimum (S3); it was also the case for 3,4-DHPEA-EDA-CARB, *p*-HPEA-EDA, pinoresinol and luteolin. Such a trend could be linked to the complex pattern of biochemical and chemical phenomena, which could affect the relative amount of phenols in the VOOs and, to a lesser extent, to the natural variability of the sampled material. 

Although no organoleptic assessment of the oils was carried out in this study, the relationship between phenolic compounds and the sensory features of the product is well known and confirmed by numerous works [5,44,52]. *p*-HPEA-EDA, also known as oleocanthal, have been proved to be the major compound responsible for pungent notes while other secoiridoid derivatives are more strongly linked to bitterness [44]. Considering the TPC (Table 3), the phenolic profile (Table 4) and the similarities with the data reported for the Coratina cultivar olive oils [11,28,53], which are well known to be strongly bitter and pungent, it might be supposed that the VOOs from the Oliva Rossa landrace have noteworthy bitter and pungent notes, although such a hypothesis should be verified by an in-depth study. To date, the few results available in the literature seem to confirm such a hypothesis [24].

The headspace volatile profile of the oils extracted in 2017/2018 is reported in Table 5.

The volatile compounds found in Oliva Rossa oils were those usually found in good-quality VOOs [4]. The most abundant ones were C5 and C6 aldehydes, alcohols and esters deriving from the well-known lipoxygenase pathway (LOX) [4], together with a remarkable amount of 3-ethyl-1,5-octadiene.

Regardless of the ripening stage of the drupes, (*E*)-2-hexenal was the most abundant compound. (*E*)-2-hexenal was reported to be strongly correlated with the bitter, almond, green, green apple-like, fatty, almond-like and cut-grass notes of the VOOs [4]. In decreasing order, 3-ethyl-1,5-octadiene was the following one. It was already detected in VOOs, although generally in quite lower concentrations [35]. The remarkable amount found in Oliva Rossa oils might be a typical trait of this olive landrace. Indeed, 3-ethyl-1,5-octadiene was already identified as one of the markers for varietal discrimination in Turkish olive oils [54]. It is worthy to note that the results reported here refer to only one crop season and thus further studies are needed to corroborate such evidence. 

During ripening, significant differences in the volatile profile were observed [4,5]. Less ripe drupes (S2) gave oils with less total volatile compounds, whose profile was dominated by (*E*)-2-hexenal, 3-ethyl-1,5-octadiene, C5 compounds (1-penten-3-one, (*Z*)-2-penten-1-ol, 1-penten-3-ol) and (*Z*)-3-hexen-1-ol. In particular, (*Z*)-3-hexen-1-ol, which was significantly higher at S2 with respect to the other stages, was already reported as one of the indicators of the early stage of ripeness [4]. 

At the technological optimum (i.e., S3), the highest amount of total volatile compounds was observed. In detail, the oils were characterized by the significantly higher content of pentanal, 3-ethyl-1,5-octadiene, 1-penten-3-one, (*E*)-2-hexenal and (*Z*)-2-penten-1-ol. It is reported that the maximum content of (*E*)-2-hexenal is reached when the drupes’ pigmentation changes from green to purple and then decrease with ripening [4,5]. However, this trend could be even cultivar dependent.

At S3, a noticeable content of acetic acid was found, too. Acetic acid could originate from the fermentation process and might be linked to some organoleptic defects such as wine-vinegary [55]. However, the detection of such defects involves many other volatile compounds [55]. At S4, a decrease in the total volatile compounds was observed with respect to S3. The oils were characterized by an increase in the acetate esters, which was associated with a decrease in the acetic acid content. A significantly higher content of 2-methyl butanal, 3-methyl butanal and 1-penten-3-ol was reported, too. The correlation among the volatile compounds and sensory features of the VOOs is well known [4,5,56]. Based on this, and considering the data reported in Table 5, it could be supposed that Oliva Rossa oils have notes of fruity, green, cut-leaves and almond, which is in accordance with the few data available in the literature [24]. 

## 4. Conclusions

Oliva Rossa is an old olive landrace from the Apulia region mostly unknown and not yet valorized. Aiming at its valorization, some useful results have been reported. First, depending on the crop year, the evolution of the drupes’ pigmentation may vary significantly and the half-varaison of the skin color could not match the technological optimum (i.e., the maximum oil content). At its optimum, the drupes had a remarkable amount of phenolic compounds. A medium to high content of oleic acid was observed although significant differences along the years were highlighted, likely due to the different climatic conditions. When a high content of PUFA was reported, there also was significant primary oxidation observed. Thus, with respect to the European limits concerning the primary oxidation markers, this aspect deserves attention, even at the technological step, which has not been considered in this study. The oils had a remarkable amount of minor compounds, which were affected by ripening. Depending on the crop year, the noteworthy levels of MUFA and TPC could lead to oils with a good stability. The phenolic profile in the considered crop year was dominated by secoiridoids derivatives, which might indicate a product with remarkable pungent and bitter notes. Similarly, based on the available data, the volatile profile was dominated by C6 and C5 compounds arising from the LOX pathway. A distinct trait seemed to be the high content of 3-ethyl-1,5-octadiene. Further investigations are needed to confirm the results obtained and to estimate the environmental effect on the oil composition of Oliva Rossa.

## Figures and Tables

**Table 1:** Fatty acid composition of the Oliva Rossa virgin olive oils (VOOs) extracted during fruit ripening in two different harvest seasons (mean ± SD, *n* = 3).

		2017/2018			2019/2020	
Pi *	1.92	1.93	3.12	2.43	3.57	3.83
Fatty acid	S2	S3	S4	S2	S3	S4
C14:0	0.01 ± 0.00 ^b,c^	0.01 ± 0.00 ^b,c^	0.01 ± 0.00 ^c^	0.01 ± 0.01 ^c^	0.02 ± 0.00 ^a,b^	0.03 ± 0.01 ^a^
C16:0	11.11 ± 0.28 ^b^	10.24 ± 0.02 ^b^	12.84 ± 0.61 ^a^	12.54 ± 0.28 ^a^	10.31 ± 0.03 ^b^	10.75 ± 0.80 ^b^
C16:1	0.48 ± 0.01 ^c^	0.48 ± 0.00 ^c^	0.55 ± 0.01 ^c^	1.04 ± 0.07 ^a^	0.78 ± 0.01 ^b^	0.79 ± 0.10 ^b^
C17:0	0.04 ± 0.00 ^d^	0.05 ± 0.00 ^c,d^	0.05 ± 0.00 ^c,d^	0.10 ± 0.01 ^a^	0.10 ± 0.01 ^a,b^	0.07 ± 0.03 ^b,c^
C17:1	0.07 ± 0.01 ^a^	0.08 ± 0.00 ^a^	0.07 ± 0.00 ^a^	0.07 ± 0.01 ^a^	0.05 ± 0.01 ^b^	0.04 ± 0.01 ^b^
C18:0	2.75 ± 0.01 ^a^	2.56 ± 0.00 ^b,c,d^	2.47 ± 0.04 ^c,d^	2.46 ± 0.10 ^d^	2.65 ± 0.03 ^a,b^	2.59 ± 0.11 ^b,c^
C18:1	73.58 ± 0.17 ^c^	76.21 ± 0.01 ^a^	74.38 ± 0.50 ^b^	71.83 ± 0.32 ^d^	70.84 ± 0.19 ^e^	70.73 ± 0.50 ^e^
C18:2	10.37 ± 0.11 ^b^	8.80 ± 0.01 ^c^	8.24 ± 0.13 ^d^	10.63 ± 0.17 ^b^	13.51 ± 0.11 ^a^	13.20 ± 0.31 ^a^
C18:3	0.77 ± 0.00 ^a^	0.77 ± 0.00 ^a^	0.65 ± 0.02 ^b^	0.57 ± 0.02 ^c^	0.61 ± 0.04 ^b,c^	0.56 ± 0.06 ^c^
C20:0	0.40 ± 0.00 ^b^	0.40 ± 0.01 ^b^	0.37 ± 0.00 ^b^	0.31 ± 0.09 ^b^	0.59 ± 0.07 ^a^	0.60 ± 0.06 ^a^
C20:1	0.38 ± 0.02 ^c^	0.37 ± 0.02 ^c^	0.36 ± 0.03 ^c^	0.32 ± 0.02 ^c^	0.46 ± 0.04 ^b^	0.54 ± 0.03 ^a^
C22:0	0.01 ± 0.00 ^b^	0.01 ± 0.00 ^b^	0.01 ± 0.00 ^b^	0.06 ± 0.03 ^a^	0.06 ± 0.03 ^a^	0.06 ± 0.02 ^a^
C24:0	0.01 ± 0.00 ^b^	0.01 ± 0.00 ^b^	0.01 ± 0.00 ^b^	0.05 ± 0.02 ^a^	0.03 ± 0.01 ^b^	0.03 ± 0.01 ^b^
SFA	14.34 ± 0.27 ^b^	13.27 ± 0.03 ^c^	15.75 ± 0.65 ^a^	15.53 ± 0.30 ^a^	13.76 ± 0.04 ^b,c^	14.13 ± 0.75 ^b,c^
MUFA	74.52 ± 0.16 ^c^	77.15 ± 0.02 ^a^	75.36 ± 0.54 ^b^	73.27 ± 0.27 ^d^	72.12 ± 0.16 ^e^	72.10 ± 0.38 ^e^
PUFA	11.14 ± 0.11 ^b^	9.57 ± 0.01 ^c^	8.89 ± 0.11 ^d^	11.20 ± 0.15 ^b^	14.12 ± 0.15 ^a^	13.76 ± 0.37 ^a^
O/L	7.09 ± 0.06 ^c^	8.66 ± 0.01 ^b^	9.03 ± 0.08 ^a^	6.76 ± 0.12 ^d^	5.24 ± 0.06 ^e^	5.36 ± 0.09 ^e^

* Pi (mean, *n* = 3) of the drupes at the moment of oil extraction; SFA, total saturated fatty acids; MUFA, total monounsaturated fatty acids; PUFA, total polyunsaturated fatty acids; O/L, oleic acid over linoleic acid ratio. Different letters for each parameter indicate significant differences according to two-way ANOVA followed by Fisher’s LSD post-hoc test (α = 0.05). S2–S4: three subsequent samplings.

**Table 2:** Basic quality parameters of the Oliva Rossa VOOs extracted during fruit ripening in two different harvest seasons (mean ± SD, *n* = 3).

Harvest Season	Pi *	Sampling	FFA (g 100 g^−1^)	PV (mEq O_2_ kg^−1^)	K_232_	K_270_
	1.92	S2	0.61 ± 0.06 ^a^	7.49 ± 0.28 ^c^	1.94 ± 0.08 ^c^	0.21 ± 0.03 ^a,b^
2017/2018	1.93	S3	0.58 ± 0.07 ^a^	7.15 ± 0.23 ^c^	1.80 ± 0.12 ^d^	0.20 ± 0.06 ^a,b^
	3.12	S4	0.58 ± 0.06 ^a^	6.05 ± 0.28 ^d^	1.76 ± 0.02 ^d^	0.23 ± 0.04 ^a^
	2.43	S2	0.52 ± 0.04 ^a,b^	8.92 ± 0.22 ^b^	2.34 ± 0.05 ^b^	0.17 ± 0.02 ^a,b^
2019/2020	3.57	S3	0.43 ± 0.06 ^b,c^	11.02 ± 0.48 ^a^	2.46 ± 0.10 ^a,b^	0.18 ± 0.03 ^a,b^
	3.83	S4	0.34 ± 0.07 ^c^	11.03 ± 0.67 ^a^	2.52 ± 0.04 ^a^	0.16 ± 0.03 ^b^

* Pi (mean, *n* = 3) of the drupes at the moment of oil extraction; FFA, free fatty acids; PV, peroxide value; K_232_ and K_270_, specific absorption at 232 and 270 nm, respectively. Different letters for each parameter indicate significant differences according to two-way ANOVA followed by Fisher’s LSD post-hoc test (α = 0.05). S2–S4: three subsequent samplings.

**Table 3:** Minor compounds (mg kg^−1^) of the Oliva Rossa VOOs extracted during fruit ripening in two different harvest seasons (mean ± SD, *n* = 3).

Harvest Season	Pi *	Sampling	TPC	α-Tocopherol	β- and γ-Tocopherols	Total Tocopherols	Carotenoids	Chlorophylls
	1.92	S2	761.64 ± 20.36 ^a^	246.20 ± 10.59 ^b^	5.28 ± 0.49 ^a,b^	251.47 ± 10.94 ^b^	57.77 ± 0.94 ^a^	54.68 ± 0.90 ^b^
2017/2018	1.93	S3	577.36 ± 15.33 ^b^	237.21 ± 4.49 ^b^	4.27 ± 0.82 ^b,c^	241.47 ± 4.82 ^b^	34.39 ± 0.30 ^c^	26.96 ± 0.10 ^d^
	3.12	S4	472.83 ± 12.60 ^c^	230.48 ± 14.03 ^b^	5.60 ± 0.73 ^a,b^	235.82 ± 13.45 ^b^	48.14 ± 0.95 ^b^	40.23 ± 0.73 ^c^
	2.43	S2	592.86 ± 72.99 ^b^	349.74 ± 5.05 ^a^	6.32 ± 0.50 ^a^	356.06 ± 4.60 ^a^	47.27 ± 1.50 ^b^	66.30 ± 1.84 ^a^
2019/2020	3.57	S3	379.60 ± 8.43 ^d^	235.79 ± 34.25 ^b^	3.39 ± 1.44 ^c^	239.18 ± 35.67 ^b^	23.42 ± 1.71 ^d^	23.13 ± 0.44 ^f^
	3.83	S4	358.67 ± 25.97 ^d^	248.33 ± 11.82 ^b^	4.34 ± 0.17 ^b,c^	252.67 ± 11.99 ^b^	25.22 ± 0.91 ^d^	25.27 ± 0.52 ^e^

* Pi (mean, *n* = 3) of the drupes at the moment of oil extraction; TPC, total phenolic compounds. Different letters for each parameter indicate significant differences according to two-way ANOVA followed by Fisher’s LSD post-hoc test (α = 0.05). S2–S4: three subsequent samplings.

**Table 4:** Identified phenolic compounds (mg GAE kg^−1^) of the Oliva Rossa VOOs extracted during fruit ripening in the 2017/2018 harvest season (mean ± SD, *n* = 3).

Compound	S2	S3	S4
3,4-DHPEA	0.33 ± 0.05 ^a,b^	0.42 ± 0.06 ^a^	0.25 ± 0.07 ^b^
*p*-HPEA	0.54 ± 0.03 ^b^	0.54 ± 0.02 ^b^	0.68 ± 0.05 ^a^
Vanillic acid	0.19 ± 0.02 ^a^	0.20 ± 0.02 ^a^	0.21 ± 0.06 ^a^
Syringic acid	0.44 ± 0.03 ^a^	0.29 ± 0.07 ^b^	0.36 ± 0.04 ^a,b^
3,4-DHPEA-EDA	62.35 ± 3.29 ^a^	25.81 ± 4.57 ^b^	17.57 ± 1.77 ^c^
3,4-DHPEA-EDA-CARB	1.64 ± 0.23 ^b^	5.65 ± 1.04 ^a^	1.31 ± 0.40 ^b^
*p*-HPEA-EDA	30.87 ± 0.93 ^a^	13.97 ± 0.78 ^c^	20.76 ± 1.40 ^b^
Pinoresinol	13.78 ± 0.28 ^a^	9.90 ± 0.69 ^c^	11.34 ± 0.51 ^b^
Luteolin	2.02 ± 0.23 ^b^	6.76 ± 0.62 ^a^	2.35 ± 2.15 ^b^
*p*-HPEA-EA	9.01 ± 0.94 ^a^	8.63 ± 1.68 ^a^	5.84 ± 1.27 ^b^
Apigenin	3.21 ± 0.33 ^a^	3.75 ± 1.27 ^a^	4.31 ± 1.50 ^a^
Total	124.38 ± 3.80 ^a^	75.92 ± 7.40 ^b^	64.98 ± 5.60 ^b^

GAE, gallic acid equivalents. 3,4-DHPEA, hydroxytyrosol; *p*-HPEA, tyrosol; 3,4-DHPEA-EDA, decarboxymethyl oleuropein-aglycone di-aldehyde; 3,4-DHPEA-EDA-CARB, carboxymethyl oleuropein-aglycone di-aldehyde; *p*-HPEA-EDA, decarboxymethyl ligstroside-aglycone di-aldehyde; *p*-HPEA-EA, ligstroside-aglycon. Different letters for each parameter indicate significant differences according to one-way ANOVA followed by Fisher’s LSD post-hoc test (α = 0.05). S2–S4: three subsequent samplings.

**Table 5:** Identified volatile compounds (mg OE kg^−1^) of the Oliva Rossa VOOs extracted during fruit ripening in the 2017/2018 harvest season (mean ± SD, *n* = 3).

Compound	S2	S3	S4
Methyl acetate	2.28 ± 0.58 ^b^	1.82 ± 0.89 ^b^	5.25 ± 0.29 ^a^
Ethyl acetate	0.40 ± 0.05 ^b^	1.25 ± 0.49 ^a^	1.47 ± 0.03 ^a^
2-Methyl butanal	1.83 ± 0.43 ^b^	1.65 ± 0.22 ^b^	5.18 ± 0.54 ^a^
3-Methyl butanal	2.53 ± 0.57 ^b^	2.07 ± 0.18 ^b^	7.84 ± 0.44 ^a^
3-Pentanone	1.25 ± 0.29 ^a^	1.64 ± 0.29 ^a^	1.69 ± 0.20 ^a^
Pentanal	1.07 ± 0.12 ^c^	13.78 ± 1.73 ^a^	4.00 ± 0.79 ^b^
3-Ethyl-1,5-octadiene	15.81 ± 3.00 ^b^	37.05 ± 9.52 ^a^	16.60 ± 2.21 ^b^
1-Penten-3-one	6.23 ± 0.67 ^c^	16.34 ± 0.84 ^a^	8.42 ± 0.78 ^b^
Hexanal	2.72 ± 0.44 ^b^	4.81 ± 0.42 ^a^	4.29 ± 0.85 ^a^
(*E*)-2-Pentenal	0.82 ± 0.19 ^a,b^	1.32 ± 0.24 ^a^	0.65 ± 0.32 ^b^
1-Penten-3-ol	2.95 ± 0.34 ^c^	4.85 ± 0.42 ^b^	10.52 ± 0.15 ^a^
(*E*)-2-Hexenal	70.37 ± 9.18 ^b^	111.88 ± 14.16 ^a^	86.69 ± 7.13 ^a,b^
(*Z*)-3-Hexen-1-yl acetate	0.84 ± 0.20 ^b^	0.10 ± 0.03 ^b^	11.42 ± 1.11 ^a^
(*Z*)-2-Penten-1-ol	4.51 ± 0.46 ^c^	18.16 ± 1.30 ^a^	9.94 ± 1.07 ^b^
Acetic acid	3.69 ± 0.36 ^b^	8.48 ± 1.19 ^a^	3.59 ± 1.44 ^b^
Hexan-1-ol	1.49 ± 0.92 ^a^	1.10 ± 0.30 ^a^	1.99 ± 0.69 ^a^
(*Z*)-3-Hexen-1-ol	4.47 ± 0.31 ^a^	1.71 ± 0.09 ^c^	3.41 ± 0.44 ^b^
Total	123.25 ± 9.81 ^b^	228.00 ± 17.31 ^a^	182.95 ± 7.97 ^a^

OE, 1-octanol equivalents. Different letters for each parameter indicate significant differences according to one-way ANOVA followed by Fisher’s LSD post-hoc test (α = 0.05). S2–S4: three subsequent samplings.

## Data Availability

Not applicable.

## Supplementary Materials

The following are available online at https://www.mdpi.com/2304-8158/10/2/369/s1, Figure S1: Monthly average temperature (°C) and rainfall (mm) recorded at the nearest weather station of Turi (Bari, Italy) for four years (2016–2019). The field including Oliva Rossa trees is about at 8 km from the weather station. Figure S2: UHPLC-DAD phenolic profile of Oliva Rossa VOOs extracted at different sampling times in 2017/2018 harvest season. IS, internal standard; (1) 3,4-DHPEA; (2) *p*-HPEA; (3) Vanillic acid; (4) Syringic acid; (5) 3,4-DHPEA-EDA; (6) 3,4-DHPEA-EDA-CARB; (7) *p*-HPEA-EDA; (8) Pinoresinol; (9) Luteolin; (10) *p*-HPEA-EA; (11) Apigenin.
