# Supplementary material for: The Potential of Apulian Olive Biodiversity: The Case of Oliva Rossa Virgin Olive Oil
Source: Foods. 2021 Feb 9;10(2):369. doi: 10.3390/foods10020369 (PMC7915085; doi:10.3390/foods10020369)
Supplement: Supplementary file 1 [file foods-10-00369-s001.pdf]

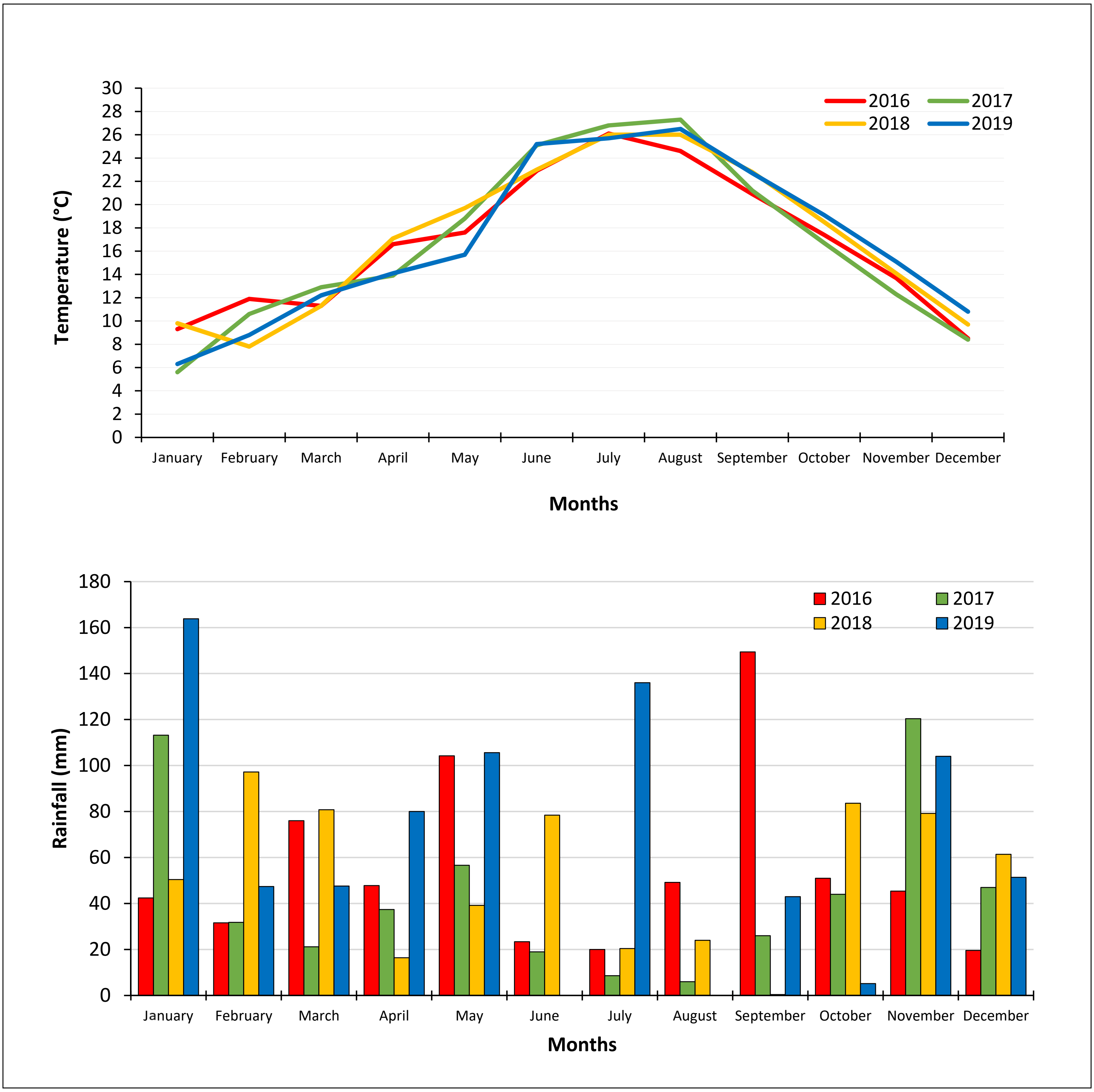

Figure S1. Monthly average temperature (°C) and rainfall (mm) recorded at the nearest weather station of Turi (Bari, Italy) for four years (2016–2019). The field including Oliva Rossa trees is about at 8 km from the weather station.

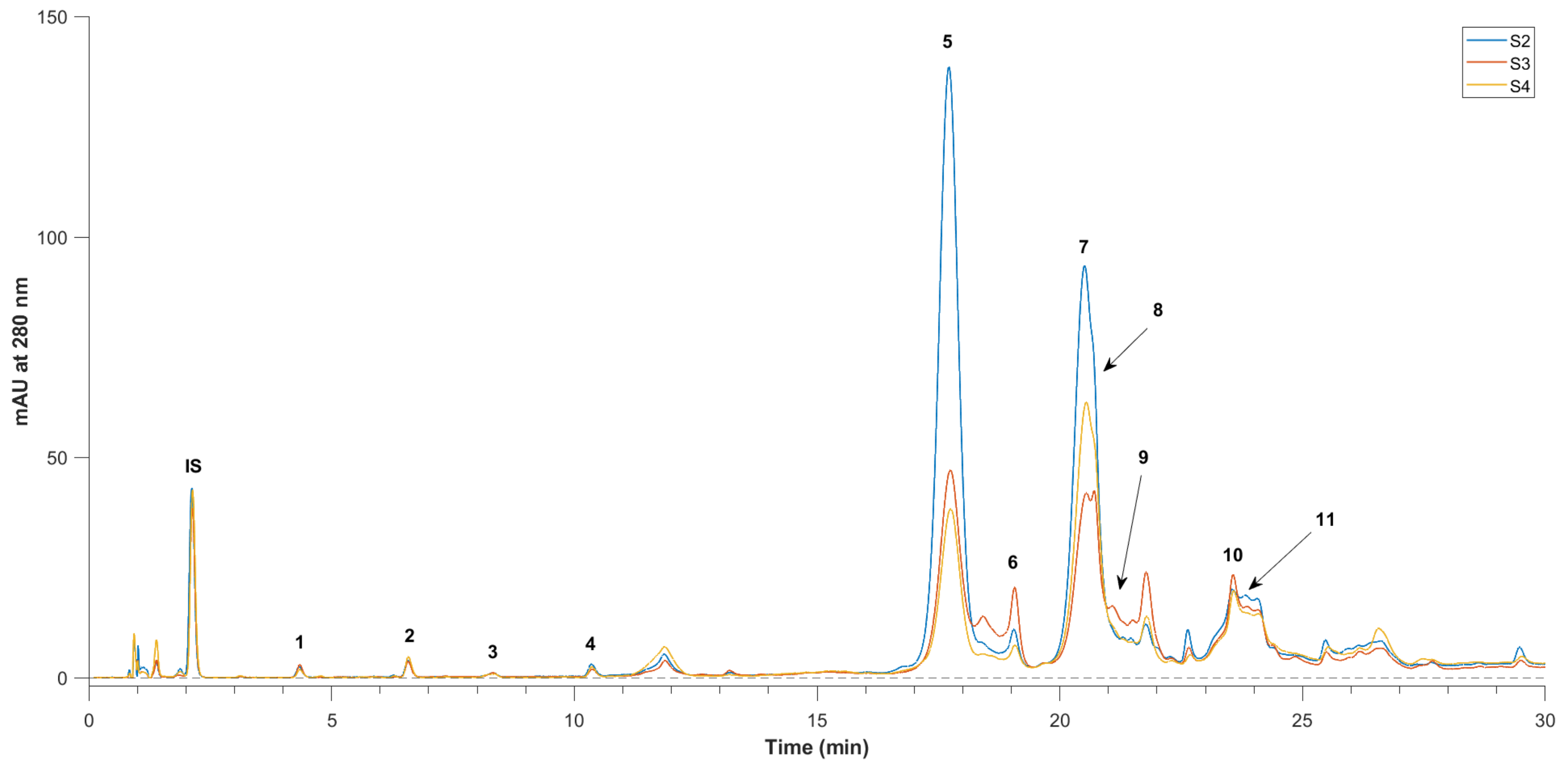

Figure S2. UHPLC-DAD phenolic profile of Oliva Rossa VOOs extracted at different sampling times in 2017/2018 harvest season. IS, internal standard; (1) 3,4-DHPEA; (2) *p*-HPEA; (3) Vanillic acid; (4) Syringic acid; (5) 3,4-DHPEA-EDA; (6) 3,4-DHPEA-EDA-CARB; (7) *p*-HPEA-EDA; (8) Pinoresinol; (9) Luteolin; (10) *p*-HPEA-EA; (11) Apigenin. S2–S4: three subsequent samplings.
